# Dynamics and Development of the COVID-19 Epidemic in the United States: A Compartmental Model Enhanced With Deep Learning Techniques

**DOI:** 10.2196/21173

**Published:** 2020-08-21

**Authors:** Qi Deng

**Affiliations:** 1 College of Economics and Management China-Africa International Business School Zhejiang Normal University Jinhua China; 2 Cofintelligence Financial Technology Ltd Shanghai China; 3 Cofintelligence Financial Technology Ltd Hong Kong Hong Kong

**Keywords:** epidemiology, COVID-19, compartmental models, deep learning, model, modeling, transmission, estimation, virus, simulate

## Abstract

**Background:**

Compartmental models dominate epidemic modeling. Transmission parameters between compartments are typically estimated through stochastic parameterization processes that depends on detailed statistics of transmission characteristics, which are economically and resource-wise expensive to collect.

**Objective:**

We aim to apply deep learning techniques as a lower data dependency alternative to estimate transmission parameters of a customized compartmental model, for the purpose of simulating the dynamics of the US coronavirus disease (COVID-19) epidemic and projecting its further development.

**Methods:**

We constructed a compartmental model and developed a multistep deep learning methodology to estimate the model’s transmission parameters. We then fed the estimated transmission parameters to the model to predict development of the US COVID-19 epidemic for 35 and 42 days. Epidemics are considered suppressed when the basic reproduction number (*R_0_*) is less than 1.

**Results:**

The deep learning–enhanced compartmental model predicts that *R_0_* will fall to <1 around August 17-19, 2020, at which point the epidemic will effectively start to die out, and that the US “infected” population will peak around August 16-18, 2020, at 3,228,574 to 3,308,911 individual cases. The model also predicted that the number of accumulative confirmed cases will cross the 5 million mark around August 7, 2020.

**Conclusions:**

Current compartmental models require stochastic parameterization to estimate the transmission parameters. These models’ effectiveness depends upon detailed statistics on transmission characteristics. As an alternative, deep learning techniques are effective in estimating these stochastic parameters with greatly reduced dependency on data particularity.

## Introduction

The coronavirus disease (COVID-19) pathogen that has ravaged China, Europe, and the United States since December 2019 is a member of the coronavirus family, which also includes the severe acute respiratory syndrome coronavirus (SARS-CoV) and Middle East respiratory syndrome–related coronavirus (MERS-CoV). In the United States, as of July 31, 2020, there have been 4,562,038 confirmed cases and 153,314 deaths of COVID-19.

The COVID-19 pandemic is still in progress, and most of the noticeable early research is descriptive in nature, focusing on reported cases to establish the baseline demographic parameters for the disease such as age, gender, health, and medical conditions in addition to the disease’s clinical manifestations, in a Chinese context. These studies include reports on demographic characteristics, epidemiological and clinical characteristics, exposure and travel history to the epicenter, and illness timelines of laboratory-confirmed cases [1-5] as well as epidemiological information on patients from social networks and local, national, and international health authorities [6]. The spread of SARS-CoV-2 outside China (eg, Iceland) is also analyzed [7], albeit to a limited extent. Concerned about the worsening situation in New York City, researchers have characterized information on the first 393 consecutive patients with COVID-19 admitted to 2 hospitals in the city [8].

Some stage-specific studies on patients with COVID-19 have also been carried out, including a single-centered, retrospective study on critically ill adult patients in Wuhan, China [9] and a retrospective, multicenter study on adult laboratory-confirmed inpatients (≥18 years of age) from 2 Wuhan hospitals, who have been discharged or have died [10].

The aim of this paper is to establish a class of extended COVID-19 compartmental models, for which the transmission parameters are estimated by a multistep, multivariate deep learning methodology.

## Methods

### COVID-19 Epidemic Modeling

There have been attempts to model the COVID-19 epidemic dynamics. These studies add a worldwide mobile dimension, reflecting a higher level of mobility and globalization in 2020 than in 2003 (SARS) and even 2013 (MERS). The SEIR (Susceptible–Exposed–Infectious–Recovered) model is used to infer the basic reproduction ratio and simulate the Wuhan epidemic [11]; it considers domestic and international air travel to and from Wuhan to other cities to forecast the national and global spread of the virus. More sophisticated models have also been developed to correlate risk levels of foreign countries with their travel exposure to China [12,13], including a stochastic dual-SEIR approach on both the Wuhan population and international travelers, to estimate how transmission varied over time from Wuhan to international destinations [13]. Simulations on the international spread of the COVID-19 after the start of the travel ban from Wuhan on January 23, 2020, have also been conducted [14], which apply the Global Epidemic and Mobility Model to a multitude of Chinese and international cities, and a SEIR variety (SLIR, Susceptible–Latent–Infectious–Recovered) to project the impact of human-to-human transmissions. To simulate the transmission mechanism itself, a Bats-Hosts-Reservoir-People network is developed to simulate potential transmission from the infection sources (ie, bats) to humans [15].

Since March 2020, with the COVID-19 outbreak winding down in China, researchers have dedicated more efforts to analyzing the effectiveness of containment measures. Mobility and travel history data from Wuhan are used to ascertain the impact of the drastic control measures implemented in China [16]. A study investigated the spread and control of COVID-19 among Chinese cities, using data on human movements and public health interventions [17]. Using contact data for Wuhan and Shanghai and contact tracing information from Hunan Province, a group of researchers built a transmission model to study the impact of social distancing and school closure [18].

### Theoretical Foundation

Compartmental models dominate epidemic modeling on COVID-19 epidemics (and previous coronavirus outbreaks), and they require detailed statistics on transmission characteristics to estimate the stochastic transmission parameters between compartments. Essentially, these models correlate factors such as geographic distances and contact intensities among heterogeneous subpopulations with gradient probability decay. Technically, transmission parameterization applies Bayesian inference methods such as Marcov Chain Monte Carlo or Gillespie algorithm [19] simulations to form probability density functions on a cross-section in order to estimate parameters for each timestep of a multivariate time series construct. These detailed statistics on transmission characteristics are economically and resource-wise expensive to collect.

We are particularly interested in extended compartmental models that cover multiple interconnected and heterogeneous subpopulations [10,15,20]. There are also some pure time series analyses on epidemic dynamics outside of mainstream compartmental modeling, for example, the AutoRegressive Integrated Moving Average approach [21] that is typically found in financial applications. Such analyses provide another perspective.

We developed a multistep, multivariate deep learning methodology to estimate the transmission parameters. We then fed these estimated transmission parameters to a customized compartmental model to predict the development of the US COVID-19 epidemic.

**Figure 1:**
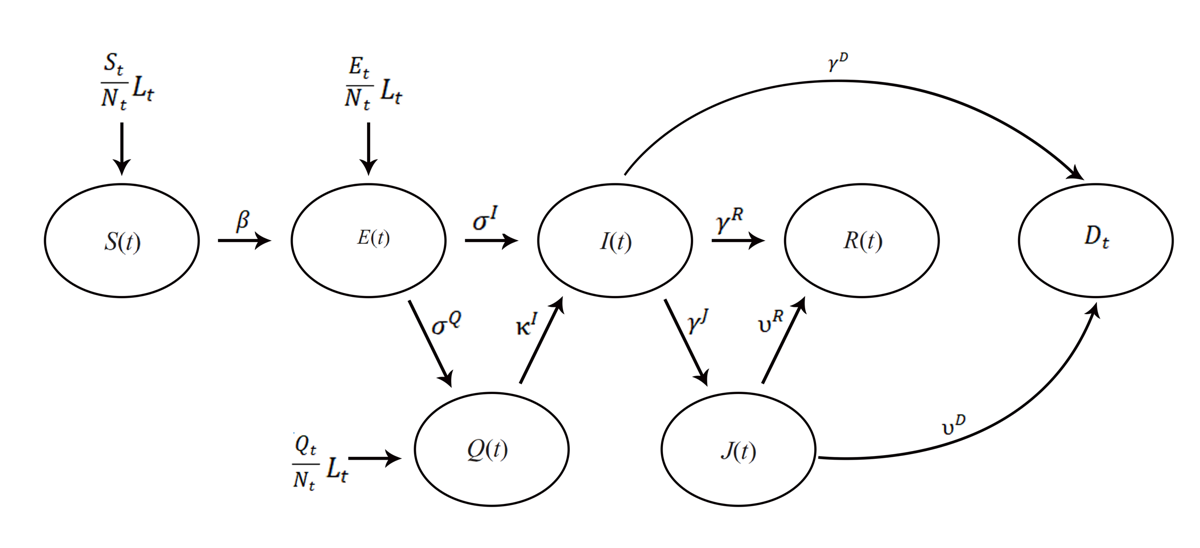
The SEIRQJD (Susceptible–Exposed–Infectious–Recovered–Quarantined–Isolated–Deceased) Model. E: Exposed; Q: Quarantined; I: Infectious; D: Deceased; S: Susceptible; I: Infectious; J: Isolated; R: Recovered. The transmission parameters (Greek letters) are - β: from Susceptible (S) to Exposed (E) if Exposed (E) is reported directly, or Susceptible (S) to Infectious (I) if Exposed (E) is not reported directly; σ^I^, σ^Q^: from Exposed (E) to Infectious (I) and Quarantined (Q), respectively; κ^I^: from Quarantined (Q) to Infectious (I); γ^J^, γ^R^, γ^D^: from Infectious (I) to Isolated (J), Recovered (R) and Deceased (D), respectively; υ^R^, υ^D^: from Isolated (J) to Recovered (R) and Deceased (D), respectively.

We established a SEIR-variety discrete time series on a daily interval as the theoretical foundation for a deep learning–enhanced compartment model. We started with the construction of a so-called SEIRQJD (SEIR-Quarantined-Isolated-Deceased) model (Figure 1).

We used the US COVID-19 epidemic datasets from John Hopkins University Center for Systems Science and Engineering (JHU CSSE) Github COVID-19 data depository, which does not include directly Exposed (E) and Quarantined (Q) data, and therefore, we set all transmission parameters to and from the “E” and “Q” compartments (σ^I^, σ^Q^, κ^I^) to 0. Furthermore, the datasets assume that all deaths arise from the isolated population (J); thus, we also set the transmission parameter from Infectious (I) to Deceased (D), γ^D^, to 0. We then simplified the SEIRJD model to a SIRJD (Susceptible–Infectious– Recovered–Isolated–Deceased) construct, in which a population is grouped into 5 compartments:

1. Susceptible (S): The susceptible population arises at a percentage 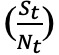 of a net influx of individuals (L_t_).
2. Infectious (I): The infectious individuals are symptomatic, come from the Susceptible compartment, and further progress into the Isolated or Recovered compartments.
3. Isolated (J): The isolated individuals have developed clinical symptoms and have been isolated by hospitalization or other means of separation. They come from the Infectious compartment and progress into the Recovered or Deceased compartments
4. Recovered (R): The recovered individuals come from Infectious and Isolated compartments and acquire lasting immunity (there is no contradiction against this assumption yet).
5. Deceased (D): The deceased cases come from the Infectious and Isolated compartments.

The SIRJD model has a daily (Δt=1) multivariate time series construct given by the follow matrix form:


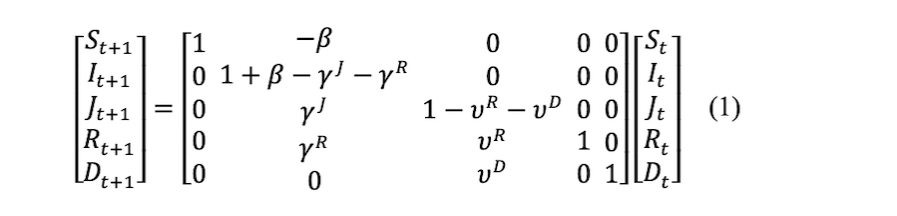


or


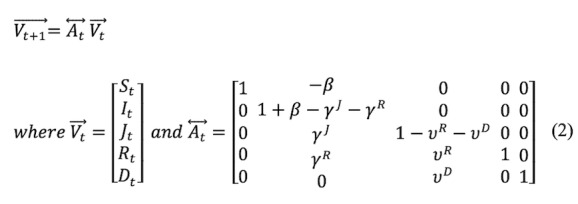


The Greek letters in the time series are transmission parameters defined in the state diagram in Figure 1. Essentially, all these parameters are stochastic.

Since we need to estimate the transmission parameters, we can rewrite and rearrange Equations (1) and (2) to the following matrix representation:


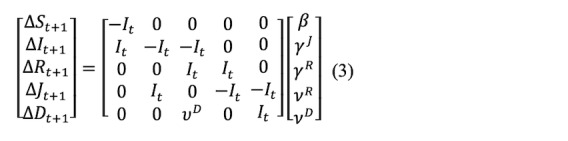


or


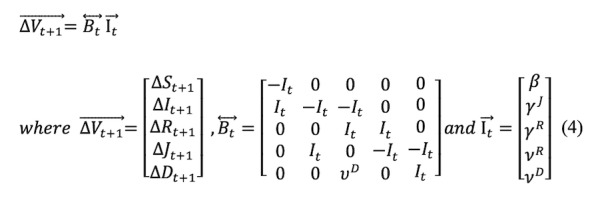


### Data

We collected the following US COVID-19 datasets from the JHU CSSE data depository [22]:

1. Dataset 1: The JHU CSSE updates daily records (confirmed, active, dead, recovered, hospitalized, etc) from April 12, 2020. We used these detailed case data to construct the compartmental model (Multimedia Appendix 1).
2. Dataset 2: The JHU CSSE updates 2 time series on a daily basis. One tracks the confirmed cases and the other tracks the dead cases, both starting from January 22, 2020. We used the confirmed/dead cases as training data for deep learning (Multimedia Appendix 2).

The JHU CSSE dataset has an almost precise period of 7 days (±1 day), indicating that a majority of the reporting agencies in the country choose to update their respective statistics on a weekly, fixed-calendar interval. We ran a 7-day moving average on the dataset to smooth out this “unnatural” data seasonality.

### Methodology

We then conducted the following step-by-step operations to model the US epidemic:

1. We constructed an in-sample SIRJD time series starting from April 12, 2020, with Dataset 1.
2. We used the in-sample SIRJD time series constructed in Step 1 to come up with an in-sample time series for the 2 most critical daily transmission parameters (β and γ^R^).
3. We constructed a confirmed/dead-case time series starting from January 22, 2020 (in-sample time series), with Dataset 2.
4. We applied 2 deep learning approaches—the standard deep neural networks (DNN) and the advanced recurrent neural networks–long short-term memory (RNN-LSTM)—to fit the confirmed/dead in-sample time series from Step 3 and predict the further development of confirmed/dead cases for 35 and 42 days (out-of-sample time series).
5. We use the confirmed/dead in-sample time series from Step 3 as training data and the in-sample β and γ^R^ time series from Step 2 as training label. We then applied the DNN and RNN-LSTM techniques to predict β and γ^R^ for 35 and 42 days (out-of-sample time series).
6. Finally, we used the predicted (out-of-sample) transmission parameters (β and γ^R^) from Step 5 to simulate 35- and 42-day progressions (out-of-sample time series) of the SIRJD model (particularly the SIR portion) in a recursive manner, starting with the data point of the last timestep from the in-sample SIRJD time series from Step 1.

**Figure 2:**
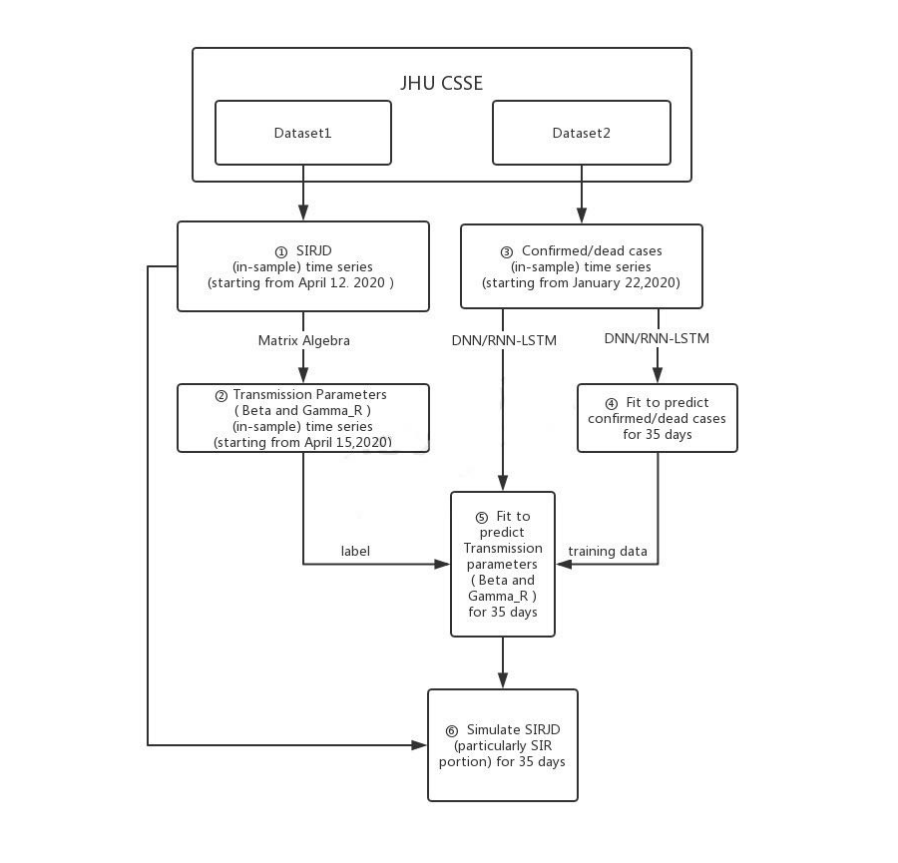
Flowchart of the dataset and methodology. JHU CSSE: John Hopkins University Center for Systems Science and Engineering; DNN: deep neural networks; RNN-LSTM: recurrent neural networks–long short-term memory; SIR: Susceptible–Infectious–Recovered; SIRJD: Susceptible–Infectious–Recovered–Isolated–Deceased.

Figure 2 presents a flowchart to illustrate the dataset and methodology.

## Results

The results based on data up to July 31, 2020, are illustrated in Figures 3-6 for the 35-day forecast and Figures 7-10 for the 42-day forecast.

**Figure 3:**
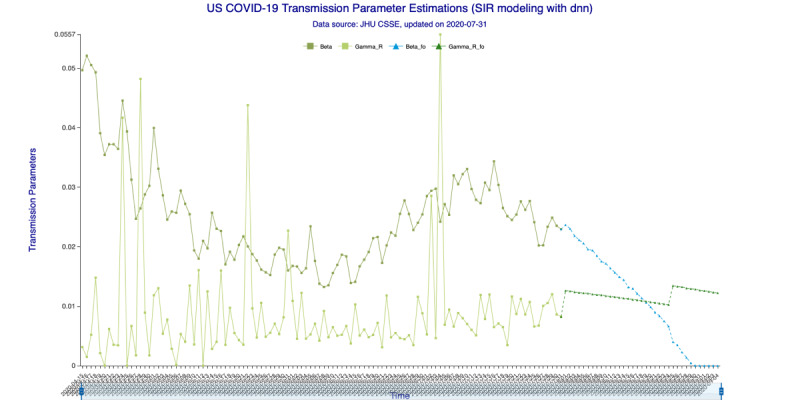
Transmission parameter estimations (deep neural networks) for 35 days. Beta is the “Susceptible-to-Infected” transmission parameter (β) and Gamma_R is the “Infected-to-Recovered” transmission parameter (γ^R^) for the in-sample (observed) data. Beta_fo is the forecasted β and Gamma_R_fo is the forecasted γ^R^ for the out-of-sample (forecasted) data.

**Figure 4:**
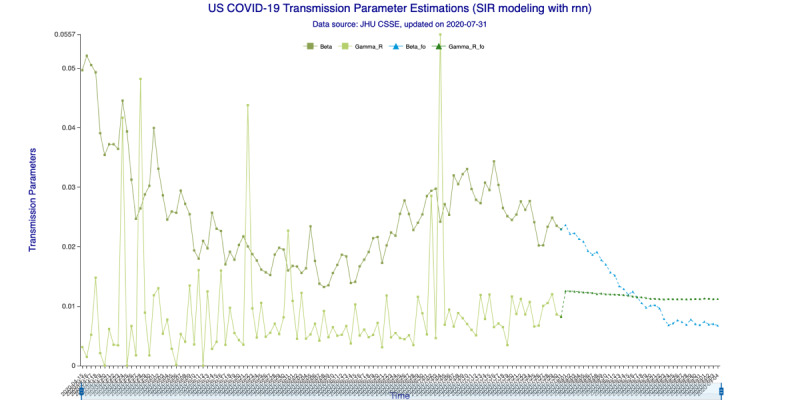
Transmission parameter estimations (recurrent neural networks–long short-term memory) for 35 days. Beta is the “Susceptible-to-Infected” transmission parameter (β) and Gamma_R is the “Infected-to-Recovered” transmission parameter (γ^R^) for the in-sample (observed) data. Beta_fo is the forecasted β and Gamma_R_fo is the forecasted γ^R^ for the out-of-sample (forecasted) data.

In Figure 3 (35-day forecast), the DNN method predicts that on August 19, 2020, the “Infected-to-Recovered” transmission parameter *γ^R^* will rise and stay above the “Susceptible-to-Infected” transmission parameter *β*. This means that the value of the basic reproduction rate, *R_0_*, will fall to <1 and that the spread of COVID-19 in the United States will effectively end on that day. In Figure 4 (35-day forecast), the RNN-LSTM method gives a slightly more aggressive prediction that *γ^R^* will overtake *β* on August 17, 2020. Thus, with the 35-day forecast, we predict that the tide of the US epidemic will turn around the August 17-19, 2020, timeframe.

**Figure 5:**
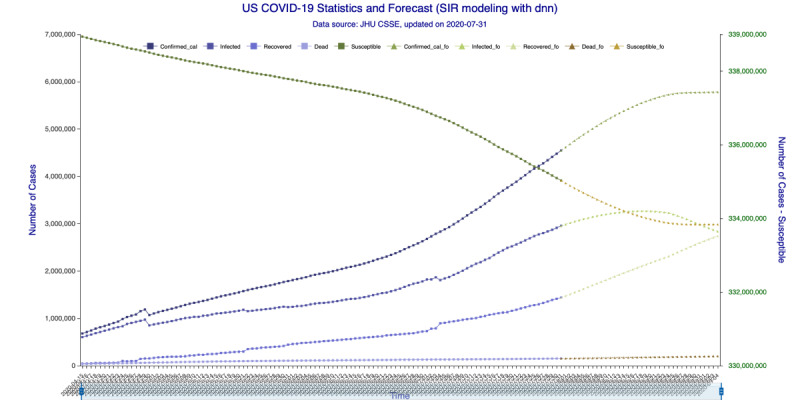
SIR (Susceptible–Infectious–Recovered) model forecasting (deep neural networks) for 35 days. Susceptible, Infected, Recovered, and Dead are in-sample compartmental model data, and Confirmed_cal is the in-sample number of confirmed cases. Susceptible_fo, Infected_fo, Recovered_fo, Dead_fo, and Confirmed_cal_fo are their out-of-sample (forecasted) counterparts. The right y-axis is for Susceptible/Susceptible_fo, while the left y-axis is for all others. The right y-axis is needed for scaling purpose, as Susceptible/Susceptible_fo are derived from the total population.

**Figure 6:**
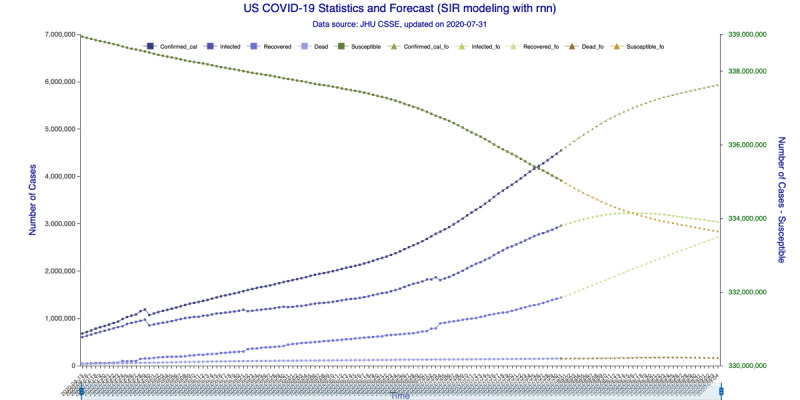
SIR (Susceptible–Infectious–Recovered) model forecasting (recurrent neural networks–long short-term memory) for 35 days. Susceptible, Infected, Recovered, and Dead are in-sample compartmental model data, and Confirmed_cal is the in-sample number of confirmed cases. Susceptible_fo, Infected_fo, Recovered_fo, Dead_fo, and Confirmed_cal_fo are their out-of-sample (forecasted) counterparts. The right y-axis is for Susceptible/Susceptible_fo, while the left y-axis is for all others. The right y-axis is needed for scaling purpose, as Susceptible/Susceptible_fo are derived from the total population.

In Figure 5 (35-day forecast), the DNN method predicts that the US “Infected” population will peak on August 18, 2020, at 3,267,907 individual cases. In Figure 6 (35-day forecast), the RNN-LSTM method predicts that the US “Infected” population will peak on August 16, 2020, at 3,228,574 individual cases. For the 35-day forecast, the deep learning methods predict that the number of accumulative confirmed cases will cross the 5 million mark on August 7, 2020, at 5,007,479 cases by DNN (Figure 5) and at 5,002,100 cases by RNN-LSTM (Figure 6).

**Figure 7:**
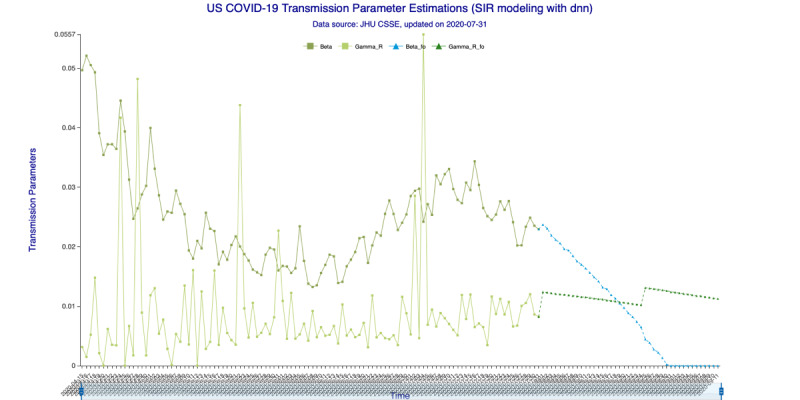
Transmission parameter estimations (deep neural networks) for 42 days. Beta is the “Susceptible-to-Infected” transmission parameter (β) and Gamma_R is the “Infected-to-Recovered” transmission parameter (γ^R^) for the in-sample (observed) data. Beta_fo is the forecasted β and Gamma_R_fo is the forecasted γ^R^ for the out-of-sample (forecasted) data.

**Figure 8:**
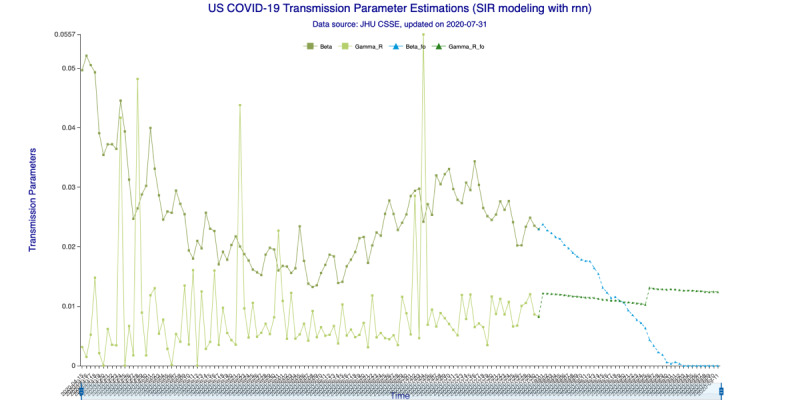
Transmission parameter estimations (recurrent neural networks–long short-term memory) for 42 days. Beta is the “Susceptible-to-Infected” transmission parameter (β) and Gamma_R is the “Infected-to-Recovered” transmission parameter (γ^R^) for the in-sample (observed) data. Beta_fo is the forecasted β and Gamma_R_fo is the forecasted γ^R^ for the out-of-sample (forecasted) data.

In Figure 7 (42-day forecast), the DNN method also predicts (same as 35-day forecast) that *γ^R^* will overtake *β* on August 19, 2020. In Figure 8 (42-day forecast), the RNN-LSTM method gives exactly the same prediction, that *R_0_* will fall to <1 on August 19, 2020.

**Figure 9:**
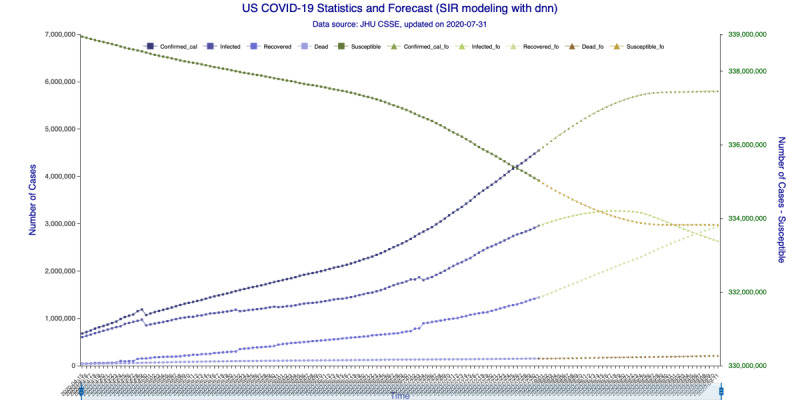
SIR (Susceptible–Infectious–Recovered) model forecasting (deep neural networks) for 42 days. Susceptible, Infected, Recovered, and Dead are in-sample compartmental model data, and Confirmed_cal is the in-sample number of confirmed cases. Susceptible_fo, Infected_fo, Recovered_fo, Dead_fo, and Confirmed_cal_fo are their out-of-sample (forecasted) counterparts. The right y-axis is for Susceptible/Susceptible_fo, while the left y-axis is for all others. The right y-axis is needed for scaling purpose, as Susceptible/Susceptible_fo are derived from the total population.

**Figure 10:**
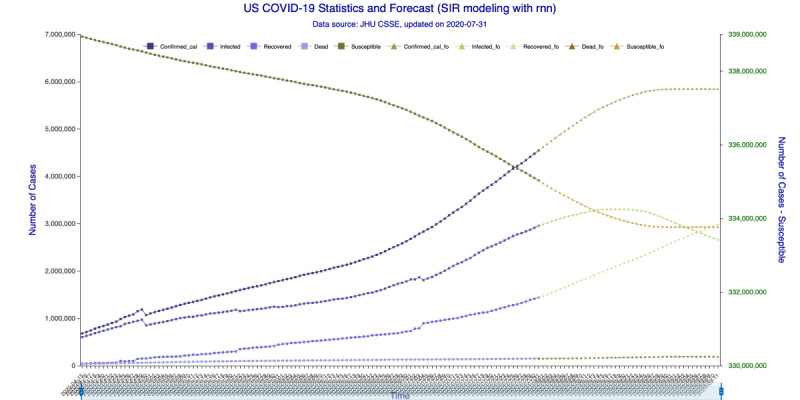
SIR (Susceptible–Infectious–Recovered) model forecasting (recurrent neural networks–long short-term memory) for 42 days. Susceptible, Infected, Recovered, and Dead are in-sample compartmental model data, and Confirmed_cal is the in-sample number of confirmed cases. Susceptible_fo, Infected_fo, Recovered_fo, Dead_fo, and Confirmed_cal_fo are their out-of-sample (forecasted) counterparts. The right y-axis is for Susceptible/Susceptible_fo, while the left y-axis is for all others. The right y-axis is needed for scaling purpose, as Susceptible/Susceptible_fo are derived from the total population.

In Figure 9 (42-day forecast), the DNN method predicts that the US “Infected” population will peak on August 18, 2020, at 3,275,304 individual cases. In Figure 10 (42-day forecast), the RNN-LSTM method predicts that the US “Infected” population will peak on August 18, 2020, at 3,308,911 individual cases. For the 42-day forecast, the deep learning methods predict that the number of accumulative confirmed cases will cross the 5 million mark on August 7, 2020, at 5,008,504 individual cases by DNN (Figure 9) and 5,014,608 individual cases by RNN-LSTM (Figure 10), which are consistent with the 35-day forecasts.

## Discussion

In this study, we applied DNN and RNN-LSTM techniques to estimate the stochastic transmission parameters for an SIRJD model with a discrete time series construct. We then used the SIRJD model to forecast further development of the US COVID-19 epidemic.

We used two US COVID-19 datasets from the JHU CSSE data depository. The first dataset includes detailed daily records (confirmed, active, dead, recovered, hospitalized, etc) starting from April 12, 2020, from which we constructed the SIRJD model. The second dataset includes time series tracked confirmed and dead cases starting from January 22, 2020, which we used to construct training data for deep learning. The JHU CSSE data have an almost precise period of 7 days (±1 day) that masks the true epidemic dynamics; thus, we ran a 7-day moving average on the dataset to smooth out this data seasonality.

We then applied DNN and RNN-LSTM deep learning techniques to fit the confirmed/dead series to predict the further development of confirmed/dead cases as well as to predict the “Susceptible-to-Infected” and “Infected-to-Recovered” transmission parameters (*β* and *γ^R^*) for 35 and 42 days. Finally, we used the predicted transmission parameters (*β* and *γ^R^*) to simulate the epidemic progression for 35 and 42 days.

With data up to July 31, 2020, the deep learning implementations predicted that the basic reproduction rate (*R_0_*) will fall to <1 around August 17-19, 2020, for the 35-day forecast and around August 19, 2020, for the 42-day forecast, at which point the spread of the coronavirus will effectively start to die out.

Implementations for the 35-day forecast predict that the US “Infected” population will peak around August 16-18, 2020, at 3,228,574 to 3,267,907 individual cases. The implementations for the 42-day forecast predict that the peak will occur on August 18, 2020, at 3,275,304 to 3,308,911 individual cases. All implementations indicate that the number of accumulative confirmed cases will cross the 5 million mark around August 7, 2020.

The 42-day forecasts provide a wider range of time and numbers than the 35-day forecasts, because for the same training data size, a longer forecast produces wider probability distributions.

With the introduction of the deep learning–enhanced compartmental model, we provide an effective and easy-to-implement alternative to prevailing stochastic parameterization, which estimates transmission parameters through probability likelihood maximization or Marcov Chain Monte Carlo simulation. The effectiveness of the prevalent approach depends upon detailed statistics on transmission characteristics among heterogeneous subpopulations, and such statistics are economically and resource-wise expensive. On the other hand, deep learning techniques uncover hidden interconnections among seemly less-related data, reducing the prediction’s dependency on data particularity. Future research on the usefulness of deep learning in epidemic modeling can further enhance its forecasting power.

## Appendix

Multimedia Appendix 1 Dataset_1.

Multimedia Appendix 2 Dataset_2.

## Abbreviations

COVID-19: coronavirus disease
DNN: deep neural networks
JHU CSSE: John Hopkins University Center for Systems Science and Engineering
MERS-CoV: Middle East respiratory syndrome–related coronavirus
RNN-LSTM: recurrent neural networks–long short-term memory
SARS-CoV: severe acute respiratory syndrome coronavirus
SEIR: Susceptible–Exposed–Infectious–Recovered
SEIRQJD: SEIR-Quarantined-Isolated-Deceased
SIRJD: Susceptible–Infectious–Recovered–Isolated–Deceased
SLIR: Susceptible–Latent–Infectious–Recovered
